# Adaptive ecological niche migration does not negate extinction susceptibility

**DOI:** 10.1038/s41598-021-94140-5

**Published:** 2021-07-29

**Authors:** A. Woodhouse, S. L. Jackson, R. A. Jamieson, R. J. Newton, P. F. Sexton, T. Aze

**Affiliations:** 1 School of Earth and Environment, University of Leeds, Leeds, LS2 9JT UK; 2 School of Environment, Earth and Ecosystem Sciences, Open University, Walton Hall, Kents Hill, Milton Keynes, MK7 6AA UK

**Keywords:** Evolutionary ecology, Palaeontology, Speciation, Biogeochemistry, Palaeoecology

## Abstract

Extinction rates in the modern world are currently at their highest in 66 million years and are likely to increase with projections of future climate change. Our knowledge of modern-day extinction risk is largely limited to decadal-centennial terrestrial records, while data from the marine realm is typically applied to high-order (> 1 million year) timescales. At present, it is unclear whether fossil organisms with common ancestry and ecological niche exhibit consistent indicators of ecological stress prior to extinction. The marine microfossil record, specifically that of the planktonic foraminifera, allows for high-resolution analyses of large numbers of fossil individuals with incredibly well-established ecological and phylogenetic history. Here, analysis of the isochronous extinction of two members of the planktonic foraminiferal genus *Dentoglobigerina* shows disruptive selection differentially compounded by permanent ecological niche migration, “pre-extinction gigantism”, and photosymbiont bleaching prior to extinction. Despite shared ecological and phylogenetic affinity, and timing of extinction, the marked discrepancies observed within the pre-extinction phenotypic responses are species-specific. These behaviours may provide insights into the nature of evolution and extinction in the open ocean and can potentially assist in the recognition and understanding of marine extinction risk in response to global climate change.

## Introduction

Current extinction rates are estimated to be at least eight times higher than the background Cenozoic (< 66 Ma) average^1^ and understanding the impacts of rapid climate change on global biodiversity is of critical importance for creating a sustainable future (https://sdgs.un.org/goals). An increasing body of evidence suggests climate state variability is potentially more important than the direction of temperature change with respect to heightened extinction rates^2,3^. As such, we look to the Cenozoic marine sedimentary record, which allows us to assess the impacts of high variability in climate state on extinct biodiversity^4,5^. In this study, we focus on the planktonic foraminifera, single-celled marine protists with a global distribution and the most complete Cenozoic species-level fossil record^6^. Their calcareous skeletons, or tests, preserve not only their entire life history, but also a biogeochemical expression of the surrounding water column (e.g.,^7^). These features allow for high-resolution species-specific quantification of physiological and ecological adaptation through periods of climate variability (e.g.,^8–21^).

Our analysis investigates the response of the planktonic foraminiferal genus *Dentoglobigerina*, of which two species (*Dentoglobigerina altispira* and *Dentoglobigerina baroemoenensis*) undergo an isochronous extinction at ~ 3.04 Ma, during a period associated with increasing climate state variability^22,23^. Through high-resolution (~ 5 kyr) paired morphometric and geochemical analyses, we demonstrate that despite the two species occupying the same ecological niche space and sharing close phylogenetic affinity, they exhibit species-specific ecological and morphological responses prior to extinction.

Morphological data enables the assessment of the relationship between body size and shape parameters likely to record long- and short-term morphometric trends in response to global climate^14^ (see Methods for designation of size and shape parameters). Single-specimen planktonic foraminiferal geochemical records allow us to place species within discrete ecological niches or “ecogroups” (see^6^), wherein the investigation of stable oxygen (δ^18^O) and carbon (δ^13^C) isotope ratios can be used to determine the relative degree of bathymetric and ecological separation within extant and extinct species^7,24^.

The methods and hypotheses tested in this study highlight the utility of the marine micropaleontological record in assessing the pre-extinction ecological response of organisms at high-resolution during intervals of global climate variability.

## Results

### Morphological records

**Figure 1:**
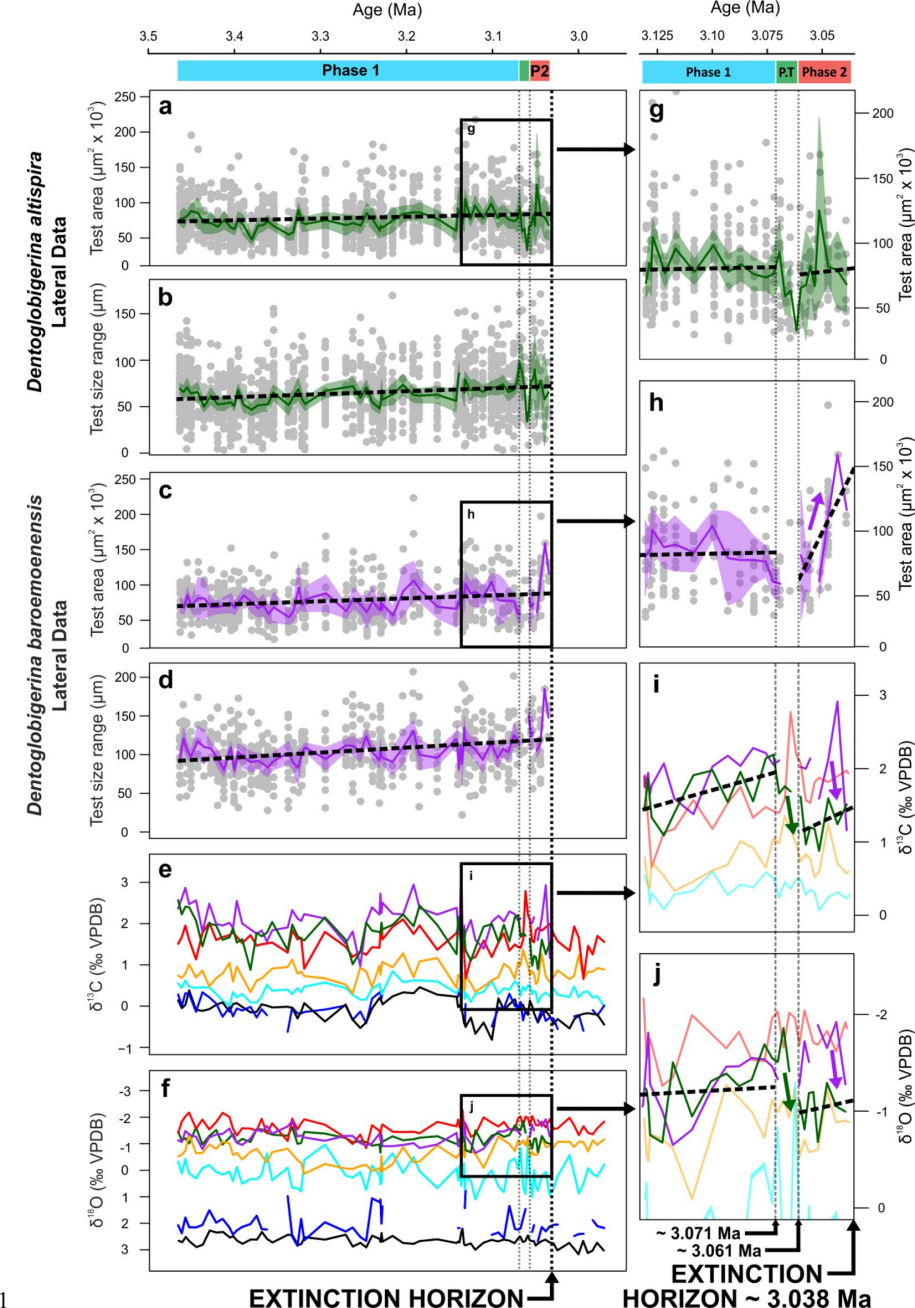
Stratigraphic log of Hole U1338A with geochemical and biotic records through dentoglobigerinid extinction event. (**a**) *D. altispira* Area, (**b**) and size range, (**c**) *D. baroemoenensis* Area, (**d**) and size range, (**e**) Single and multi-specimens planktonic foraminiferal δ^13^C, (**f**) Single and multi-specimen planktonic foraminiferal δ^18^O, (**g**–**j**) blown up dentoglobigerinid Area, and isotope data in pre-extinction interval. Black = bottom-water*,* dark blue = subthermocline, cyan = thermocline, orange = subsurface*,* red = surface mixed layer, green = *D. altispira*, purple = *D. baroemoenensis.* Solid coloured lines for dentoglobigerinids are mean values of multiple single specimen analyses, shaded areas are 95% confidence intervals, black dashed lines are species trendlines, light vertical dotted lines indicate boundaries between “Phases”, black vertical dotted line indicates extinction horizon. *P2* Phase 2, *P.T* Phase Transition.

**Figure 2:**
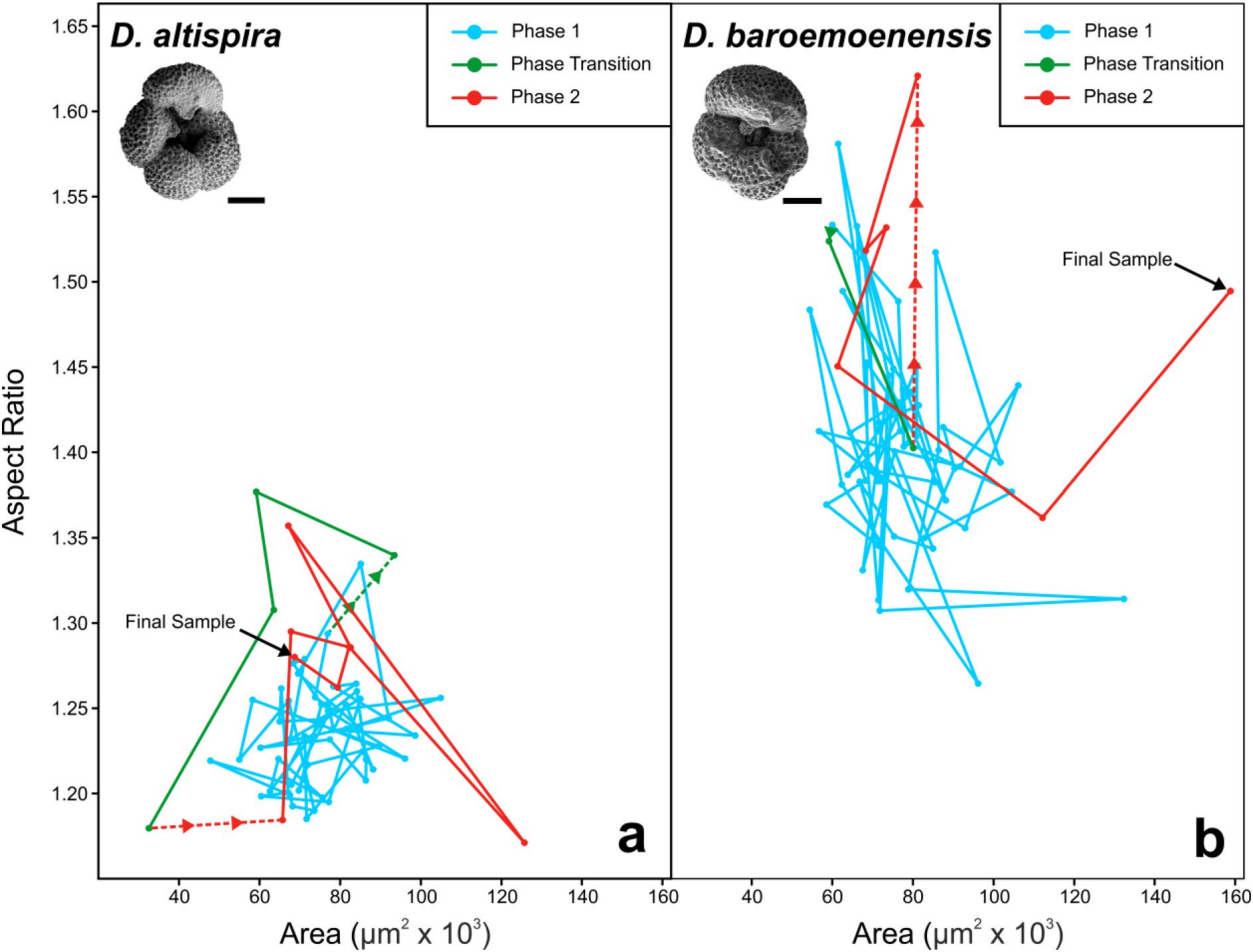
Sample means of lateral area and aspect ratio of (**a**) *D. altispira,* and (**b**) *D. baroemoenensis*. Blue = Phase 1, green = Phase Transition, Red = Phase 2. Initiation of the phase transition is defined by the excursion in *D. altispira* morphometric data (see "Morphological Records"). Initiation of Phase 2 is defined *D. altispira* morphometric data returning to Phase 1 variability (see "Morphological Records"), and additionally by geochemical excursions in *D. altispira* stable isotope data (see "Geochemical Records", Fig. 1). Scale bars = 100 μm.

There is a long-term (~ 400 kyr) morphological trend approaching the dentoglobigerinid extinction interval (~ 3.038 Ma), where both species demonstrate a general increase in body size and range (Fig. 1). At ~ 3.071 Ma (Fig. 1), ~ 30 kyrs prior to the extinction of *D. altispira,* mean shape parameters indicate a deviation from relative morphological uniformity (Fig. S1), whereby the relationship between test area and aspect ratio (Fig. 2a) shows distinct changes due to a marked decrease in mean test area (Fig. 1a). This morphological excursion ends 10 kyrs later (3.061 Ma), where the size/shape values return to background values that were more typical prior to ~ 3.071 Ma (Figs. 1, 2). We designate the respective sedimentary intervals preceding and succeeding these two morphological events to signify distinct ecological “Phases” in dentoglobigerinid pre-extinction response, herein termed “Phase 1” and “Phase 2”, respectively. Additionally, the 10 kyr interval encompassed by the two Phases is deemed to represent a “Phase Transition” (Fig. 1).

Within *D. baroemoenensis*, the size/shape relationship generally tends to show higher inter-sample variability than *D. altispira* throughout the record (Figs. 1, 2). However, the most significant changes are seen following the Phase Transition, where there is marked increase in mean test size through the final ~ 10 kyrs prior to extinction (Figs. 1, 2).

### Geochemical records

**Figure 3:**
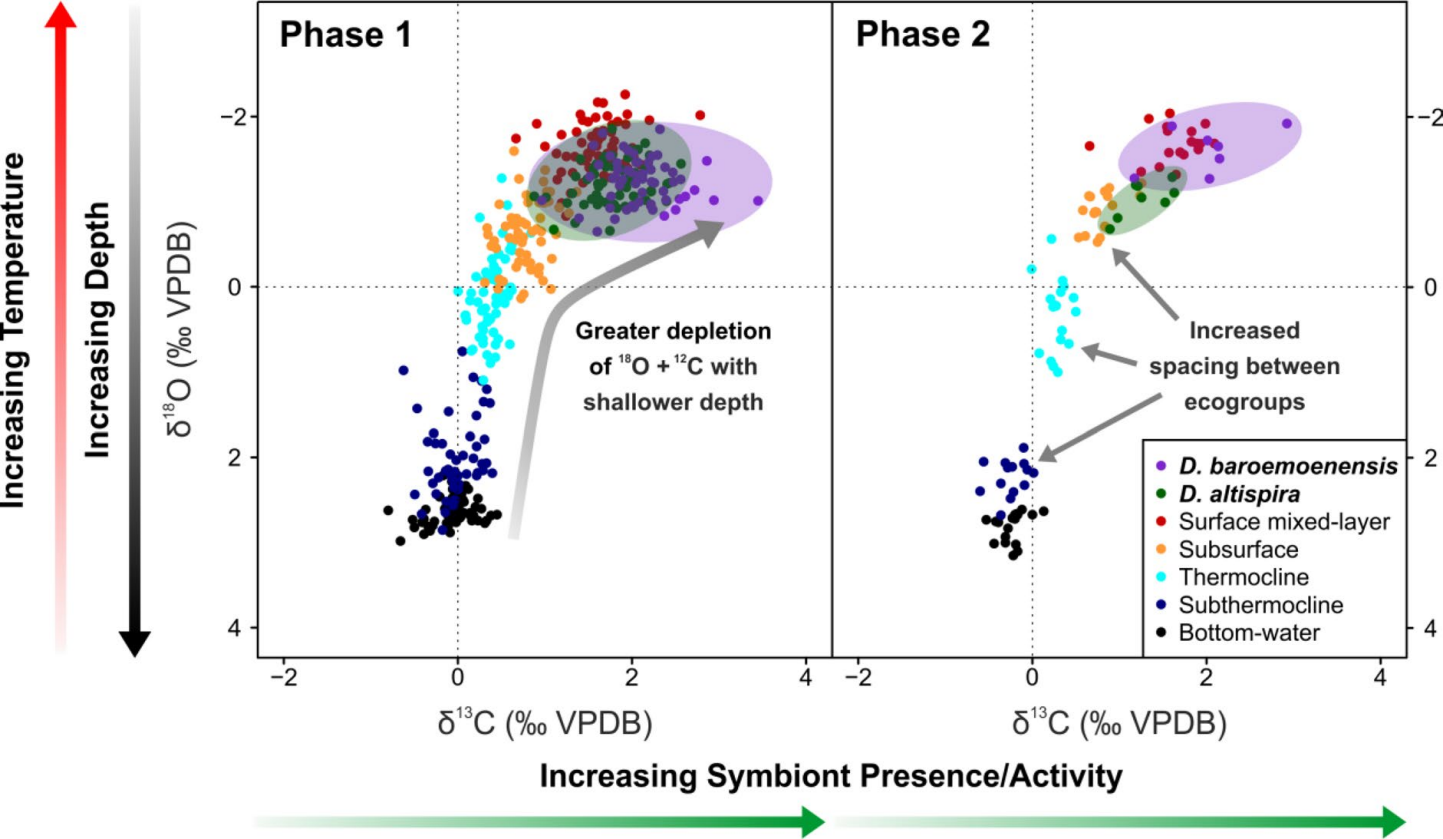
δ^13^C and δ^18^O cross-plots through Phase 1 and Phase 2. Black = Bottom-water*,* dark blue = subthermocline, cyan = thermocline, orange = subsurface*,* red = surface mixed layer, green = *D. altispira*, purple = *D. baroemoenensis. Dentoglobigerina* measurements are single specimens, others are multiple specimens.

Generally, dentoglobigerinid specimens exhibit stable isotope values typical of modern symbiont-hosting surface mixed-layer dwellers^24^ (Fig. 3, SI), with high δ^13^C and low δ^18^O, respectively. At ~ 3.061 Ma, contemporaneous with the initiation of Phase 2 (Figs. 1, 2), *D. altispira* δ^13^C and δ^18^O signals exhibit significant, permanent negative and positive shifts, respectively, to values more consistent with species living in the subsurface, rather than the surface mixed-layer (Fig. 1i, j). This signal is not reflected by *D. baroemoenensis* at this time, and it is only in the final sample prior to extinction (~ 3.038 Ma) that a substantial negative δ^13^C, and positive δ^18^O excursion consistent with the occupation of a deeper living-depth is seen in this species (Fig. 1i, j).

### Statistical analyses

Our data and linear models (see SI, Tables S1–4) identify trends in dentoglobigerinid morphology and ecology that support the designation of distinct ecological “Phases” in the final ~ 30 kyrs of our *Dentoglobigerina* species. Phase 1 is typified by stable morphological and geochemical background conditions from 3.466–3.071 Ma (Figs. 1, 2). At this point the Phase Transition commences, identified by the morphological excursion of *D. altispira* (Figs. 1, 2). Finally, 10 kyr later Phase 2 initiates, marked by the coeval end of the period of diminished size in *D. altispira*, and the geochemical excursion representing a shift in its ecological niche (Figs. 1, 2, 3). For *D. baroemoenensis,* samples sourced from the Phase Transition are notably lacking in specimens, and enhanced size increase and morphological stochasticity is observed through much of Phase 2, (Figs. 1, 2).

Models tested whether the abiotic conditions of the paleo-water column (signified by the stable isotopic signature of the present extant species with known living depths^6,24^), responded in or out of tandem with the dentoglobigerinid signature. Throughout Phase 1 the dentoglobigerinids have isotopic signals that are consistent with a surface mixed-layer species which hosts photosymbiotic algae^6,24^ (Figs. 1, 3) and the two dentoglobigerinid species δ^18^O signals respond in tandem (Tables S1 & S2). In Phase 2, there is a marked decoupling in the δ^13^C signal of the two species (Tables S3 & S4) potentially indicating a disruption of photosymbiosis for *D. altispira,* but not for *D. baroemoenensis,* which maintains its ecological affinity up to the sample preceding extinction (Fig. 1i, j). Additional linear models testing the relationship between dentoglobigerinid morphology and environment show that as bottom water δ^18^O becomes more positive and δ^13^C more negative (e.g., as mean global temperature decreases/ice extent increases) dentoglobigerinid test area increases throughout Phase 1 (Tables S1 & S2). However, for *D. altispira*, this signal is lost in Phase 2, and for *D. baroemoenensis* the signal switches to increased test area mirroring more negative bottom-water δ^18^O (Tables S3 & S4).

Grubbs’ test^25^ results (Table 1) indicate that outlier samples with statistical significance tend to be grouped within either the Phase Transition or Phase 2 for *D. altispira*, whereas all significant *D. baroemoenensis* outliers are represented by the final sample. Mann–Whitney U test^26^ results (Table 2) show a significant difference between the *D. altispira* δ^13^C signal of Phases 1 and 2. For *D. baroemoenensis*, Mann–Whitney U and z-test results (Table 2) indicate that the δ^18^O signature, and the umbilical and lateral test area and size range records are significantly different between the two phases.

**Table 1:** Grubbs' Test results.

Parameter	*D. altispira*	Position	*D. baroemoenensis*	Position
Carbon	0.5492	Within P1	0.07428	Within P1
Oxygen	0.2239	Within PT	0.7708	Within P2
U_Area	**< *0.01***	***Within P2***	**< *0.01***	***Final sample***
U_Aspect Ratio	0.1084	Within PT	0.09192	1st PT sample
U_Dmax	0.07362	Within P2	**< *0.01***	***Final sample***
U_Dmin	**< *0.05***	***Within P2***	**< *0.05***	***Final sample***
U_Range	0.2757	Within P1	0.1778	Within P2
U_Roundness	**< *0.05***	***Within P1***	NA	NA
U_Circularity	0.2506	1st PT sample	NA	NA
L_Area	**< *0.01***	***Within P2***	0.06507	Final sample
L_Aspect_Ratio	**< *0.05***	***Within PT***	0.06467	1st P2 sample
L_Dmax	0.05313	Within P2	**< *0.05***	***Final sample***
L_Dmin	**< *0.01***	***Within P2***	0.264	Penultimate sample
L_Range	0.06445	1st PT sample	0.08086	1st P2 sample
L_Roundness	**< *0.05***	***Last PT sample***	NA	NA
L_Circularity	0.562	Within P2	NA	NA

Bold and italic results indicate statistical significance (p < 0.05). U, Umbilical; L, Lateral; P1, Phase 1; P2, Phase 2; PT, Phase Transition.

**Table 2:** Mann–Whitney U test results for between *D. altispira* and *D. baroemoenen*sis phase populations.

Parameter	*D. altispira*		*D. baroemoenensis*	
	Test	P1vP2	Test	P1vP2
Carbon	*M-WU*	**< *0.01***	z-test	0.655
Oxygen	M-WU	0.183	z-test	**< *0.01***
U_Area	M-WU	0.227	M-WU	**< *0.01***
U_Aspect_Ratio	M-WU	0.806	M-WU	0.243
U_Size_Range	M-WU	0.557	M-WU	**< *0.01***
U_Roundness	M-WU	0.135	NA	NA
U_Circularity	M-WU	0.937	NA	NA
L_Area	M-WU	0.429	M-WU	**< *0.01***
L_Aspect_Ratio	M-WU	0.272	z-test	0.476
L_Size_Range	M-WU	0.227	z-test	**< *0.01***
L_Roundness	M-WU	0.506	NA	NA
L_Circularity	M-WU	0.176	NA	NA

Bold, italic results indicate statistical significance (p < 0.05). U, Umbilical; L, Lateral; P1, Phase 1; P2, Phase 2.

## Discussion

The Cenozoic planktonic foraminiferal fossil record documents a strong positive correlation between test size and the degree of global marine latitudinal and vertical temperature gradients^22,27^, however the largest intraspecific test sizes tend not to be analogous with species’ ecological optima^28,29^. In Phase 1, dentoglobigerinid test size data shows a gradual relative increase through time (Figs. 1; S1) likely representing a response to the development of temperature gradients associated with the intensification of northern hemisphere icesheets^22,23^. At the initiation of the Phase Transition (~ 3.071 Ma) stepwise disruptions in both morphology and geochemical signatures are likely indicative of disruptive selection and “bet-hedging”^30,31^, a typical response to the propagation of terminal stress levels preceding extinction^32,33^. Previous studies^15,16^ document increasing growth asymmetry and morphological trait variance as responses to abiotic forcing, wherein species produce offspring with high inter-individual phenotypic variability during unfavourable environmental conditions to improve mean population fitness^15,34–36^.

In the case of *D. altispira*, the ecological end-result following the Phase Transition approaching the termination of the record appears to be permanent ecological niche migration from the surface mixed-layer down to the subsurface, supported by Mann–Whitney U test results (Table 2) on δ^13^C signals between Phases 1 and 2 (Figs. 1, 3). Significantly, *D. baroemoenensis* displays an almost total absence during the Phase Transition (Fig. 1), supporting the prevalence of environmental conditions detrimental to dentoglobigerinid ecology. Upon its return, it shows a dramatic increase in lateral and umbilical area (Figs. 1; S1), where Mann–Whitney U test and z-test results indicate significantly different body size and range to Phase 1 (Table 2). These changes, which we term “pre-extinction gigantism”, are antithetical to the “pre-extinction dwarfing”^10,37^ previously documented in several other species, and may represent a response to the steepening of vertical and latitudinal water column temperature gradients associated with cryosphere development^22,23^, typified by more distinct spacing between the geochemically assigned ecological habits during Phase 2 (Fig. 3).

The minor general trend of increasing body size range in both species (Fig. 1a, c) potentially infers long-term mitigation of external environmental pressures expressed through rising polymorphism. Further research is required; however, such behaviour may be characteristic of temporally long ranging species^38^ when subjected to global climate state variations which deviate far from their ancestral ecosystem^39,40^.

The study species share close phylogenetic and ecological affinity^38^, maintain high-order morphological likeness from speciation, and undergo isochronous extinction, yet the phenotypic responses recorded prior to extinction are species-specific. Rapid within-clade character change, cladogenesis, and extinction during periods of detrimental environmental change are likely common-place within the history of life^32^, and phylogenetically and ecologically adjacent taxa can exhibit similarities in selection pressures which do not necessarily trigger an adaptive response in the same direction^32,33,39^.

For *D. altispira*, migration from the surface mixed layer to the subsurface may be compounded either by a photosymbiont reduction/suppression^18^, or adoption of a facultative symbiotic ecological strategy, recognized to enhance flexibility of nutritional sources through minimal energetic investment^41,42^. This proposed adaptation is suggested over total algal photosymbiont “bleaching”^9,11,18,21,43^, as *D. altispira* continues to present δ^13^C enrichment higher than values observed in the asymbiotic, subsurface-dwelling taxa analysed in this study^42^, yet similar δ^18^O values (Figs. 1, 3).

For *D. baroemoenensis*, water column temperature gradient dynamics associated with thermocline shallowing^22,44^ are a potential trigger for the brief vacation and apparent “pre-extinction gigantism” exhibited through Phase 2. The final sample of this species’ record may be marked by symbiont bleaching wherein, despite a size increase, specimens exhibit a reduction in δ^13^C values (Fig. 1). Alternatively, *D. baroemoenensis* may also be recording rapid migration down to the subsurface, or a significant change in the extent of test calcification just prior to extinction^24,45,46^.

Photosymbiont bleaching driven by extreme heat stress has been recorded in extant groups such as corals^47^ and larger benthic foraminifera^48,49^, and previously, records of the potential bleaching of algal photosymbionts within fossil planktonic foraminifera have been confined to early Cenozoic hyperthermals (e.g.,^9,11,18,21,43^). Despite bleaching amongst modern corals being primarily driven by increasing temperatures^47,50,51^, a multitude of environmental stressors are associated with bleaching responses^52,53^, and the prospective bleaching of *D. baroemoenensis* may indicate that this pre-extinction response in symbiont-bearing taxa may be more common than previously thought during intervals not characterised by elevated temperatures.

Model results (Tables S1–4) lend further support to our interpretations, in which both morphological and ecological responses display discrete signatures between phases, characteristic of disruptive selection^30,31^. The interpreted behaviour of these organisms raises some interesting questions. One of the most pressing and fundamental issues for palaeoceanography, is whether fossil organisms identified via their external morphology which are used for the inference of paleoclimatic data maintain ecological uniformitarianism for the entirety of their stratigraphic range. Our study, alongside other novel research on modern and fossil populations^18,54–63^ suggests not, and as such deriving environmental interpretations from fossil taxa, particularly during intervals of climate variability, should be treated with caution. Whether the documentation of these behaviours indicate failed efforts at stress mitigation via water-depth associated parapatric anagenesis is currently undetermined, but further high-resolution comparable investigations through speciation events may help to understand the fundamental mechanisms driving evolution and extinction in an ecosystem with limited vicariance potential such as the open ocean.

## Summary

The studied section exhibits a high-resolution record of the pre-extinction biotic response of two members of the planktonic foraminiferal genus *Dentoglobigerina* during major global palaeoceanographic changes associated with the development of northern hemisphere ice sheet formation. Despite the species’ phylogenetic and ecological affinities, documented phenotypic responses are species-specific, wherein both *D. altispira* and *D. baroemoenensis* exhibit evidence of permanent adaptive ecological niche migration and photosymbiont reduction. In addition, *D. baroemoenensis* documents “pre-extinction gigantism”, and potential photosymbiont bleaching. This study highlights the importance of high-resolution analyses when investigating biological responses and extinction dynamics. The unparalleled resolution of the marine microfossil record allows us to identify and evaluate past occurrences of morpho-ecological stochasticity indicative of disruptive selection and niche adaptation. However more comprehensive studies utilising multiple localities are required to improve our understanding and identification of the potential for pre-extinction signals to better recognise extinction risk in response to rapid climate change.

## Methods

### Site selection

Material was sourced from Integrated Ocean Drilling Program Expedition 321 Site U1338 (Hole 1338A) (2°30.469′N, 17°58.162′W) situated in the East Equatorial Pacific, which was drilled to 410.0 mbsf through Holocene-early Miocene pelagic sediments^64^. At ~ 3 Ma, the site was in a deep-water pelagic environment of similar water depth and paleolatitude^65^ to the modern. The primary lithologies represented are calcareous, diatom and radiolarian nannofossil oozes and chalks. Despite the deep-water settings and primarily calcareous nature of the sediments, excellent microfossil preservation has been recorded throughout this core interval^66^. A preliminary assessment of core U1338A was carried out to determine the approximate position of the extinction of the dentoglobigerinids (~ 3 Ma) based on tropical biostratigraphy^67^, and shipboard paleomagnetic data^64^.

### Assemblage analysis

Sediment volumes of 20–40 cc were collected and washed with de-ionised water over a 63-µm sieve; the residues were dried in an oven at 40 °C and split. All samples were examined using a Zeiss Stemi 305 Compact Stereo Microscope. We identified planktonic foraminifers following the taxonomy of Kennett and Srinivasan^68^, Schiebel and Hemleben^27^, and Wade et al.^38^ and performed assemblage counts on 300 individuals from > 63 µm splits.

### Chronology determination

During sampling of the extinction interval and identification of the dentoglobigerinid extinction event, specimens of *Cibicidoides wuellerstorfi* were also picked to create a benthic foraminiferal δ^18^O record. This record was constructed and tuned to the Ocean Drilling Program Site 849/IODP Site 1338 stack constructed by Lyle et al.^69^ using QAnalySeries software^70^ to better constrain the timing of pre-extinction responses compared to using palaeomagnetic data alone.

### Morphometrics and repeatability

The first 50 (where present) complete specimens of the genus *Dentoglobigerina* were picked and mounted in umbilical position on card slides pierced with a fine needle to accommodate the variably spired nature of species in the genus^38^. Specimens were imaged umbilically using a Zeiss Axio Zoom V16 microscope with attached Canon EOS 100D camera at × 19.4 magnification. All specimens were then rotated 90° laterally, and imaged whilst propped onto their penultimate chamber. Images were processed using the image analysis software Image Pro Premier, and the “size” trait parameters: test area (Area, µm^2^), and test size range (minimum test diameter (Dmin)–maximum test diameter (Dmax)), and “shape” trait parameters: aspect ratio (AR, ratio between maximum test height and width), roundness (perimeter^2^ (µm)/4π.test area), and circularity (4.test area)/(π.MaxFeret^2^), were captured from both orientations, extracted, and databased (see SI). To determine whether the size and shape parameters were repeatably valid measurements, trait repeatability^71,72^ was performed by removing, remounting, and reimaging 200 specimens of each analysed dentoglobigerinid species (100 umbilical orientations, and 100 lateral). Measurements of the repeated runs (Fig. S2) are plotted using continuous frequency distributions (kernel density estimates with a Gaussian kernel and bandwidth *h* = 1.06*sn^1/5^ following Silverman^73^, with *s* the standard deviation of trait measurements per species and *n* the number of analysed individuals). Results were evaluated through Wilcoxon signed-rank test using R software^74^. Where mean run rank differences deviated significantly, measured traits were deemed non-repeatable (Fig. S3). All measured size and shape trait parameters were deemed repeatable for *D.* altispira, whereas for *D. baroemoenensis*, roundness and circularity were not repeatable, and were subsequently removed from further interpretations. Repeatable traits were then subjected to power analysis^75^ using the ‘pwr’ package in R^76^ to determine the minimum number of individuals required to detect mean sample trait changes of 5, 10, 15, 20, 25, and 30% with power > 0.9 and a significance level of p = 0.01 as suggested by Brombacher et al.^71^ (Fig. S4; Table S5).

### Stable isotope analysis

For stable isotope analysis, species-specific size fractions were screened and picked for exceptionally preserved specimens of *Dentoglobigerina altispira* (> 200 µm)*,* and *Dentoglobigerina baroemoenensis* (> 200 µm) exhibiting “excellent” and “glassy” preservation^77^*.* The same screening process was performed for nominate taxa representing specific ecological habits through the water column: *Globigerinoides ruber* (212–350 µm, surface mixed-layer), *Neogloboquadrina incompta* (212–350 µm, subsurface), *Globorotalia tumida* (> 300 µm, thermocline/photic zone base, corrected for a 1.0‰ δ^13^C enrichment due to this species occupying the shallow oxygen minimum zone and consequential effects of reduced ambient pH^24^), *Hirsutella scitula* (212–300 µm, subthermocline), and *Cibicidoides wuellerstorfi* (> 212 µm, bottom-water) (see^6,24,78,79^). Single specimens of dentoglobigerinids, and multiple specimens of all other foraminifer species were analysed using an Elementar IsoPrime Dual-Inlet Isotope Ratio Mass Spectrometer in the Cohen Geochemistry Laboratory, University of Leeds, and data are reported to the Vienna Pee Dee belemnite (VPDB) scale using an Elemental Microanalysis Carrera marble standard where analytical precision was better than 0.07 and 0.13 ‰ for δ^13^C and δ^18^O (1 standard deviation), respectively (Figs. 1, 3; see SI).

### Statistical analysis

Prior to statistical modelling, all sample mean isotopic and morphometric measurements were log standardised and the sample first differences generated per time step. All statistical analyses and linear modelling were carried out using R software^74^. For linear models, statistically significant relationships were identified between variables, and residual standard mean errors (RSE) were calculated to determine the model fit (Tables S3 & S4). The normality of dentoglobigerinid morphometric and geochemical parameter data was assessed using a Shapiro–Wilk test^80^ to determine whether parametric or non-parametric tests were applicable. Dentoglobigerinid sample-mean morphological and geochemical parameters were tested to identify study section outliers through Grubb’s test^25^ using the ‘outliers’ package^81^. Comparisons between all dentoglobigerinid morphological and geochemical parameters between the two phases (i.e., specimens before and after the defined Phase Transition) were performed by Mann–Whitney U^26^ using the ‘asht’ package^82^, and z-tests using the ‘BDSA’ package^83^.

## Supplementary Information

Supplementary Information 1.

Supplementary Figure 1.

Supplementary Figure 2.

Supplementary Figure 3.

Supplementary Figure 4.

Supplementary Information 6.

Supplementary Information 7.

Supplementary Information 8.

Supplementary Information 9.

Supplementary Information 10.

Supplementary Information 11.
