# Supplementary figures and images for: Adaptive ecological niche migration does not negate extinction susceptibility
Source: Sci Rep. 2021 Jul 29;11:15411. doi: 10.1038/s41598-021-94140-5 (PMC8322071; doi:10.1038/s41598-021-94140-5)

*Dentoglobigerina altispira*

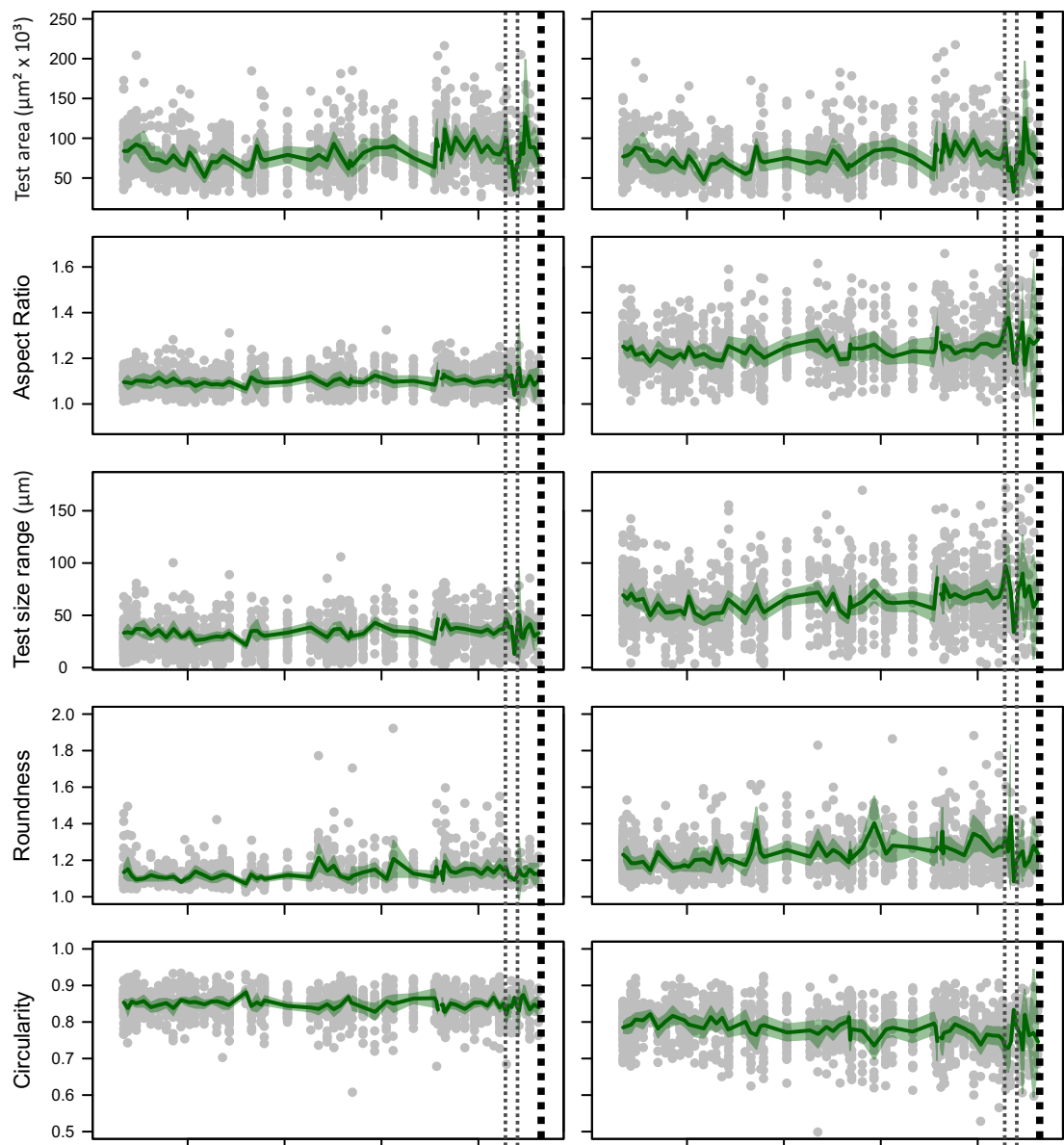

*Dentoglobigerina baroemouensis*

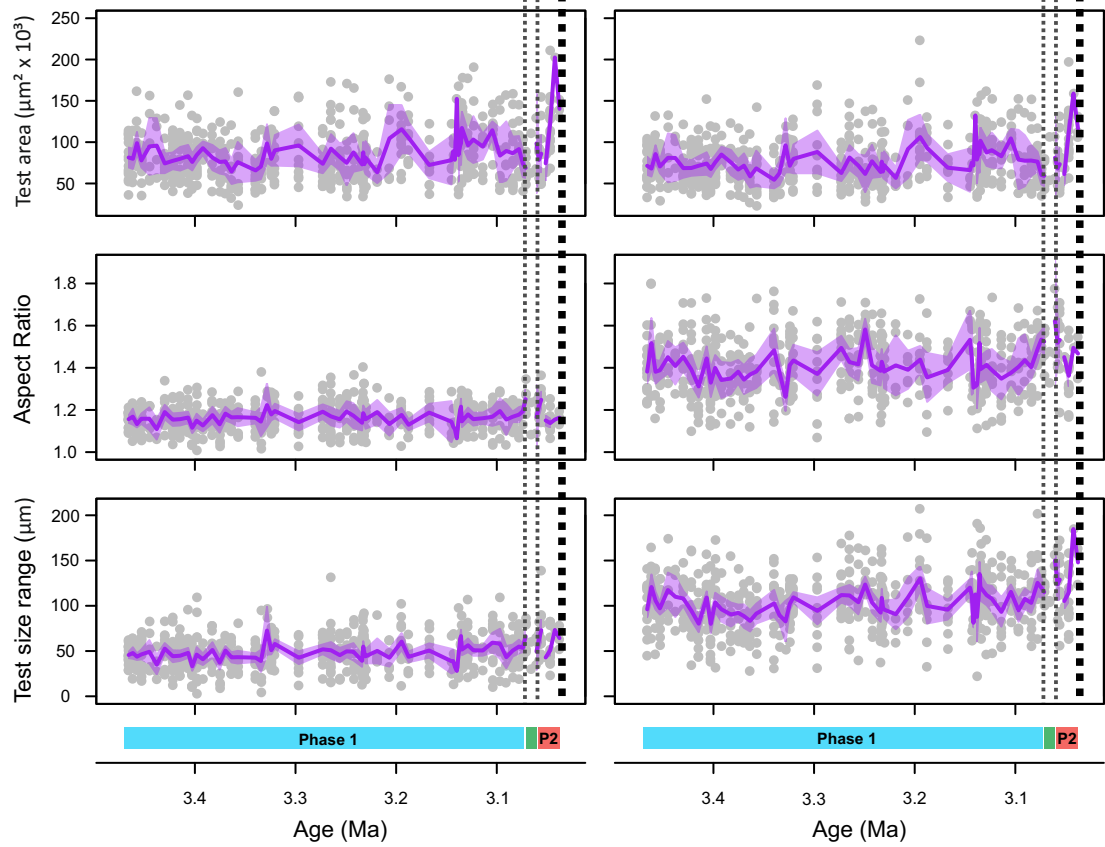

Umbilical

Lateral

Supplement: Supplementary file 2 — Supplementary Figure 1. [file 41598_2021_94140_MOESM2_ESM.pdf]

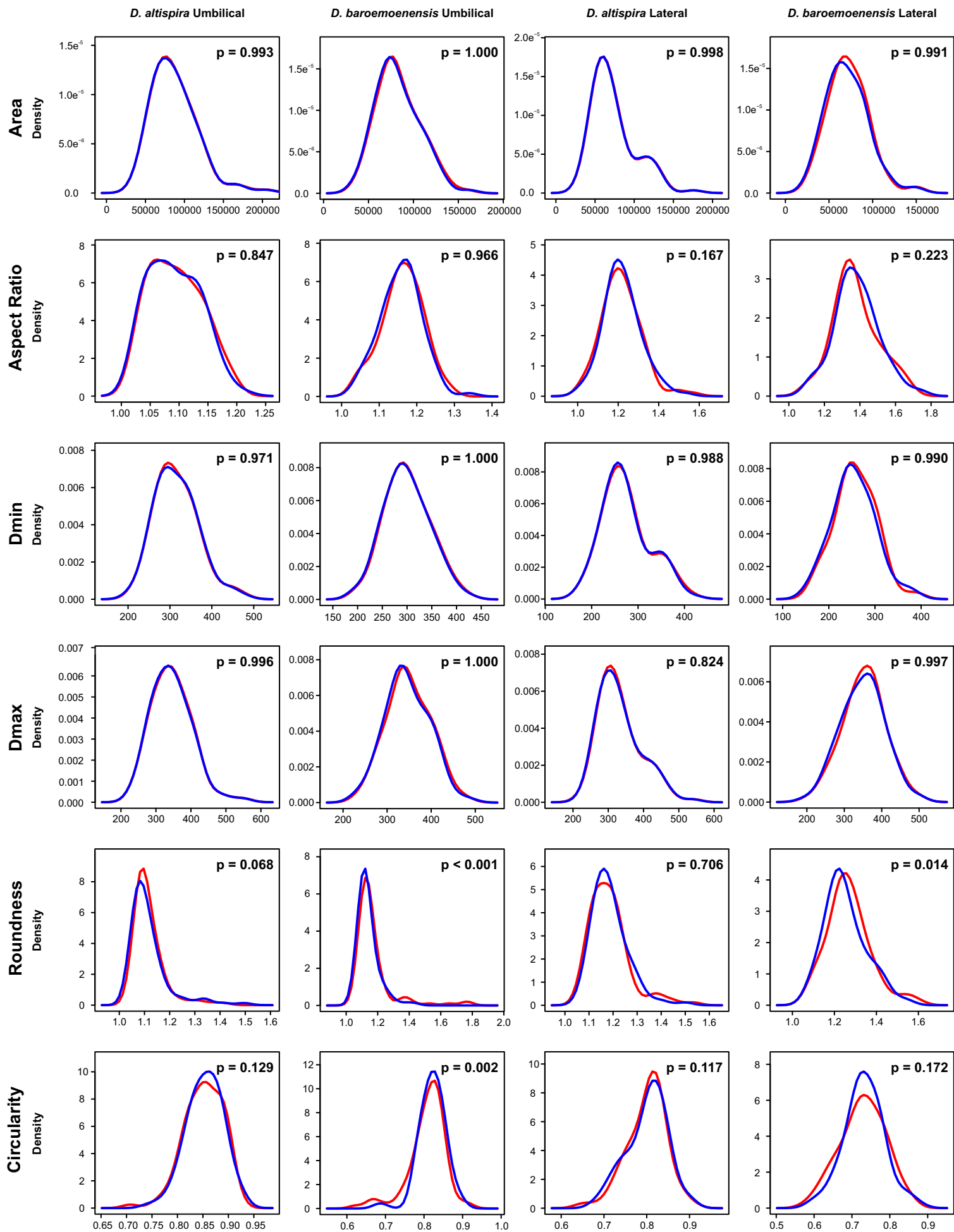

Supplement: Supplementary file 3 — Supplementary Figure 2. [file 41598_2021_94140_MOESM3_ESM.pdf]

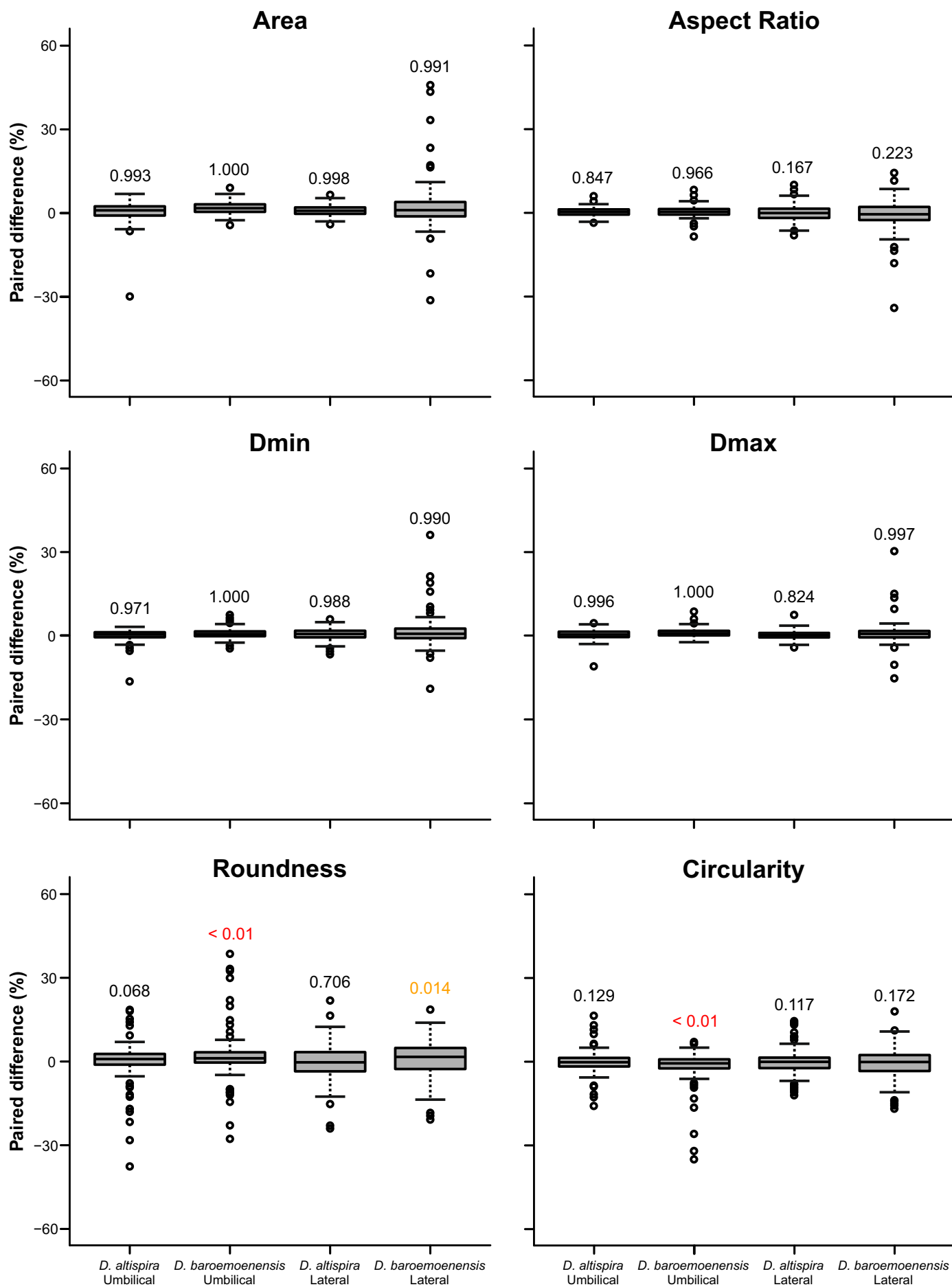

Supplement: Supplementary file 4 — Supplementary Figure 3. [file 41598_2021_94140_MOESM4_ESM.pdf]

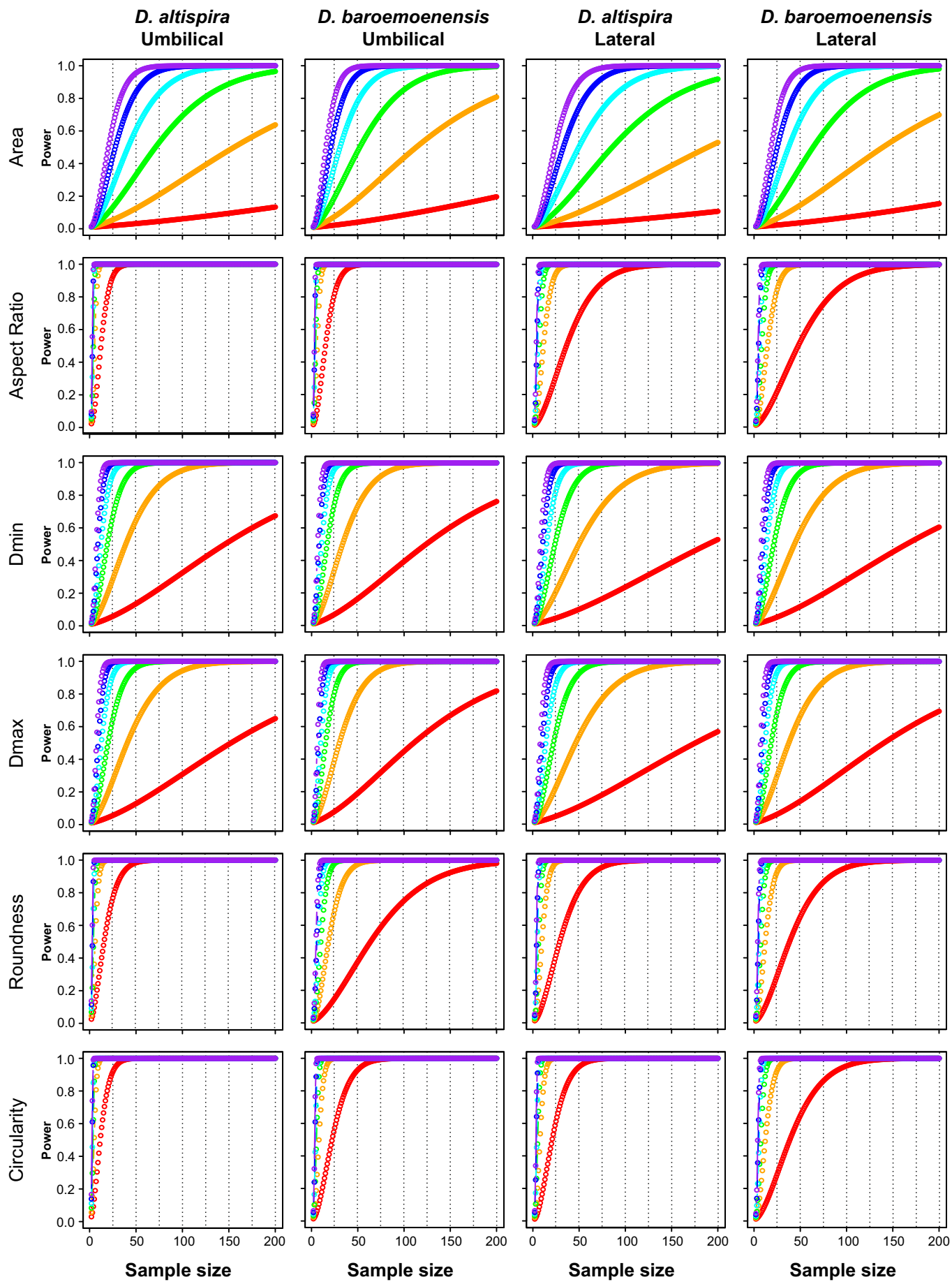

Supplement: Supplementary file 5 — Supplementary Figure 4. [file 41598_2021_94140_MOESM5_ESM.pdf]
