# Supplementary material for: Adaptive ecological niche migration does not negate extinction susceptibility
Source: Sci Rep. 2021 Jul 29;11:15411. doi: 10.1038/s41598-021-94140-5 (PMC8322071; doi:10.1038/s41598-021-94140-5)
Supplement: Supplementary file 6 — Supplementary Information 6. [file 41598_2021_94140_MOESM6_ESM.docx]

**Adaptive ecological niche migration does not negate extinction susceptibility**

A.D. Woodhouse^1^*, S.L. Jackson^1^, R.A. Jamieson^1^, R.J. Newton^1^, P.F. Sexton^2^, and

T. Aze^1^.

1. School of Earth and Environment, University of Leeds, Leeds, LS2 9JT, UK
2. School of Environment, Earth and Ecosystem Sciences, Open University, Walton Hall, Kents Hill, Milton Keynes, MK7 6AA, UK

*e-mail correspondence: eeadw@leeds.ac.uk

**Supplementary Information File**

Contains supplementary figure captions and tables.

Figure S1. Measured morphometric traits of dentoglobigerinid species. Raw values are plotted as grey dots, mean values as lines and 95% confidence intervals as shaded areas, light vertical dotted lines indicate boundaries between “Phases”, black vertical dotted line indicates extinction horizon.

Figure S2. Kernel density plots of first (red) and second (blue) set of morphometric measurements on *Dentoglobigerina altispira* and *Dentoglobigerina baroemoenensis* in umbilical and lateral orientations.

Figure S3. Barplots of the difference between repeated measurements on Area, Aspect Ratio, minimum diameter (Dmin), maximum diameter (Dmax), Roundness, and Circularity on the same individual (paired difference) expressed as percentage of the individual’s trait mean. p-values of the Wilcoxon signed-rank test performed on subsequent measurements on the same individuals are given, with p-values < 0.01 & < 0.05 indicating significant differences shown in red and orange, respectively.

Figure S4. Calibration of the number of induvial specimens required to detect a given trait change in specific orientations. Power is plotted against the number of individuals needed to detect changes in trait values by 5% (red), 10% (orange), 15% (green), 20% (cyan), 25% (blue), and 30% (magenta). Significance is set to p = 0.01.

| Table S1. *Dentoglobigerina altispira* LM results for Phase 1 | |  |  |
| --- | --- | --- | --- |
| ***Dentoglobigerina altispira* LMs** |  |  |  |
| Geochemistry vs. geochemistry |  |  |  |
| *D. altispira* ecological signal | Ecological signal | RSE | Correlation |
| Carbon | *G. tumida* δ^13^C | 28.79 | Positive |
| Carbon | *N. incompta* δ^13^C | 28.86 | Positive |
| Oxygen | *D. baroemoenensis* δ^18^O | 226.06 | Positive |
| Morphology vs. geochemistry |  |  |  |
| Trait | Ecological signal | RSE | Correlation |
| Umbilical Area | *D. baroemoenensis* δ^18^O | -53.58 | Positive |
| Umbilical Area | *C. wuellerstorfi* δ^13^C | -51.82 | Negative |
| Umbilical Area | *C. wuellerstorfi* δ^18^O | -53.35 | Positive |
| Umbilical Area | *G. tumida* δ^13^C | -54.22 | Positive |
| Lateral Area | *D. baroemoenensis* δ^18^O | -47.06 | Positive |
| Lateral Area | *C. wuellerstorfi* δ^13^C | -45.70 | Negative |
| Lateral Area | *C. wuellerstorfi* δ^18^O | -47.39 | Positive |
| Umbilical Aspect Ratio | *H. scitula* δ^13^C | -28.32 | Positive |
| Lateral Aspect Ratio | *N. incompta* δ^18^O | 29.23 | Negative |
| Lateral Range | *N. incompta* δ^18^O | -23.37 | Negative |
| Umbilical Circularity | *G. ruber* δ^13^C | 23.27 | Negative |
| Lateral Circularity | *N. incompta* δ^18^O | 18.69 | Positive |
| *D. altispira* Morphometry vs. Geochemistry |  |  |  |
| *D. altispira* ecological signal | Trait | RSE | Correlation |
| Carbon | Umbilical Area | 26.54 | Positive |
| Carbon | Lateral Area | 26.83 | Positive |
| Carbon | Umbilical Aspect Ratio | NA |  |
| Carbon | Lateral Aspect Ratio | NA |  |
| Carbon | Umbilical Range | 27.23 | Positive |
| Carbon | Lateral Range | 27.86 | Positive |
| Carbon | Umbilical Roundness | NA |  |
| Carbon | Lateral Roundness | NA |  |
| Carbon | Umbilical Circularity | NA |  |
| Carbon | Lateral Circularity | NA |  |
| Oxygen | Umbilical Area | NA |  |
| Oxygen | Lateral Area | NA |  |
| Oxygen | Umbilical Aspect Ratio | NA |  |
| Oxygen | Lateral Aspect Ratio | NA |  |
| Oxygen | Umbilical Range | NA |  |
| Oxygen | Lateral Range | NA |  |
| Oxygen | Umbilical Roundness | NA |  |
| Oxygen | Lateral Roundness | NA |  |
| Oxygen | Umbilical Circularity | NA |  |
| Oxygen | Lateral Circularity | NA |  |

| Table S2. *Dentoglobigerina baroemoenensis* LM results for Phase 1 | | | |  |  | |
| --- | --- | --- | --- | --- | --- | --- |
| ***Dentoglobigerina baroemoenensis* LMs** | | |  |  |  | |
| Geochemistry vs. geochemistry | | |  |  |  | |
| *D. baroemoenensis* ecological signal | | | Ecological signal | RSE | Correlation | |
| Carbon | | | *N. incompta* δ^13^C | 116.58 | Negative | |
| Carbon | | | *N. incompta* δ^18^O | 117.42 | Negative | |
| Carbon | | | *G. ruber* δ^18^O | 117.11 | Positive | |
| Oxygen | | | *D. altispira* δ^18^O | 71.98 | Positive | |
| Morphology vs. geochemistry | | |  |  |  | |
| Trait | | | Ecological signal | RSE | Correlation | |
| Umbilical Area | | | *C. wuellerstorfi* δ^13^C | 64.56 | Negative | |
| Umbilical Area | | | *C. wuellerstorfi* δ^18^O | 66.34 | Positive | |
| Lateral Area | | | *C. wuellerstorfi* δ^13^C | 57.93 | Negative | |
| Umbilical Aspect Ratio | | | *N. incompta* δ^13^C | -22.61 | Negative | |
| Lateral Aspect Ratio | | | *H. scitula* δ^13^C | -32.66 | Negative | |
| Lateral Aspect Ratio | | | *G. tumida* δ^13^C | -30.27 | Negative | |
| Lateral Aspect Ratio | | | *G. ruber* δ^18^O | -30.88 | Positive | |
| Umbilical Range | | | *N. incompta* δ^13^C | -31.94 | Negative | |
| Lateral Range | | | *G. tumida* δ^13^C | -47.80 | Negative | |
| Lateral Range | | | *G. ruber* δ^13^C | -48.21 | Negative | |
| Lateral Range | | | *G. ruber* δ^18^O | -48.40 | Positive | |
| *D. baroemoenensis* Morphometry vs. Geochemistry | | |  |  |  | |
| *D. baroemoenensis* ecological signal | | | Trait | RSE | Correlation | |
| Carbon | | | Umbilical Area | 107.00 | Positive | |
| Carbon | | | Lateral Area | 106.66 | Positive | |
| Carbon | | | Umbilical Aspect Ratio | NA | NA | |
| Carbon | | | Lateral Aspect Ratio | NA | NA | |
| Carbon | | | Umbilical Range | NA | NA | |
| Carbon | | | Lateral Range | 107.74 | Positive | |
| Oxygen | | | Umbilical Area | NA | NA | |
| Oxygen | | | Lateral Area | NA | NA | |
| Oxygen | | | Umbilical Aspect Ratio | NA | NA | |
| Oxygen | | | Lateral Aspect Ratio | 69.012 | Negative | |
| Oxygen | | | Umbilical Range | NA | NA | |
| Oxygen | | | Lateral Range | NA | NA | |
|  | | |  |  |  | |
| Table S3. *Dentoglobigerina altispira* LM results for Phase 2 | | |  | |  |  |
| ***Dentoglobigerina altispira* LMs** |  | |  | |  |  |
| Geochemistry vs. geochemistry |  | |  | |  |  |
| *D. altispira* ecological signal | Ecological signal | | RSE | | Correlation |  |
| Carbon | *H. scitula* δ^13^C | | 6.11 | | Positive |  |
| Carbon | *H. scitula* δ^18^O | | 4.28 | | Negative |  |
| Oxygen | *G. tumida* δ^13^C | | -13.89 | | Negative |  |
| Morphology vs. geochemistry |  | |  | |  |  |
| Trait | Ecological signal | | RSE | | Correlation |  |
| Umbilical Range | *G. ruber* δ^18^O | | -38.31 | | Negative |  |
| Umbilical Aspect Ratio | *G. ruber* δ^18^O | | -72.35 | | Negative |  |
| Lateral Circularity | *H. scitula* δ^13^C | | 1.02 | | Negative |  |
| Umbilical Roundness | *N. incompta* δ^18^O | | -6.15 | | Negative |  |
| Umbilical Roundness | *G. ruber* δ^18^O | | -5.69 | | Negative |  |
| Lateral Roundness | *D. baroemoenensis* δ^13^C | | -3.04 | | Positive |  |
| *D. altispira* Morphometry vs. Geochemistry |  | |  | |  |  |
| *D. altispira* ecological signal | Trait | | RSE | | Correlation |  |
| Carbon | Umbilical Area | | NA | | NA |  |
| Carbon | Umbilical Aspect Ratio | | NA | | NA |  |
| Carbon | Umbilical Range | | NA | | NA |  |
| Carbon | Umbilical Roundness | | NA | | NA |  |
| Carbon | Umbilical Circularity | | NA | | NA |  |
| Carbon | Lateral Area | | NA | | NA |  |
| Carbon | Lateral Aspect Ratio | | NA | | NA |  |
| Carbon | Lateral Range | | NA | | NA |  |
| Carbon | Lateral Roundness | | NA | | NA |  |
| Carbon | Lateral Circularity | | NA | | NA |  |
| Oxygen | Umbilical Area | | NA | | NA |  |
| Oxygen | Umbilical Aspect Ratio | | NA | | NA |  |
| Oxygen | Umbilical Range | | NA | | NA |  |
| Oxygen | Umbilical Roundness | | NA | | NA |  |
| Oxygen | Umbilical Circularity | | NA | | NA |  |
| Oxygen | Lateral Area | | NA | | NA |  |
| Oxygen | Lateral Aspect Ratio | | NA | | NA |  |
| Oxygen | Lateral Range | | NA | | NA |  |
| Oxygen | Lateral Roundness | | NA | | NA |  |
| Oxygen | Lateral Circularity | | NA | | NA |  |

| Table S4. *Dentoglobigerina baroemoenensis* LM results for Phase 2 | |  |  |
| --- | --- | --- | --- |
| ***Dentoglobigerina baroemoenensis* LMs** |  |  |  |
| Geochemistry vs. geochemistry |  |  |  |
| *D. baroemoenensis* ecological signal | Ecological signal | RSE | Correlation |
| Carbon | *C. wuellerstorfi* δ^18^O | 3.07 | Negative |
| Oxygen | *D. altispira* δ^13^C | 360.66 | Positive |
| Morphology vs. geochemistry |  |  |  |
| Trait | Ecological signal | RSE | Correlation |
| Umbilical Area | *C. wuellerstorfi* δ^18^O | -2.43 | Negative |
| Lateral Area | *G. tumida* δ^13^C | -3.58 | Positive |
| Lateral Area | *C. wuellerstorfi* δ^18^O | -1.69 | Negative |
| Lateral Area | *C. wuellerstorfi* δ^13^C | -3.21 | Positive |
| *D. baroemoenensis* Morphometry vs. Geochemistry |  |  |  |
| *D. baroemoenensis* ecological signal | Trait | RSE | Correlation |
| Carbon | Umbilical Area | 3.23 | Positive |
| Carbon | Lateral Area | NA | NA |
| Carbon | Umbilical Aspect Ratio | NA | NA |
| Carbon | Lateral Aspect Ratio | NA | NA |
| Carbon | Umbilical Range | NA | NA |
| Carbon | Lateral Range | NA | NA |
| Oxygen | Umbilical Area | NA | NA |
| Oxygen | Lateral Area | NA | NA |
| Oxygen | Umbilical Aspect Ratio | NA | NA |
| Oxygen | Lateral Aspect Ratio | NA | NA |
| Oxygen | Umbilical Range | NA | NA |
| Oxygen | Lateral Range | NA | NA |

| Table S5. Power analysis results for dentoglobigerinid size and shape parameters indicating no. of specimens required to detect % change | | | | | | |
| --- | --- | --- | --- | --- | --- | --- |
|  |  |  |  |  |  |  |
| ***D. altispira*** | **% Parameter Change** | | | | | |
| **Umbilical** | **5** | **10** | **15** | **20** | **25** | **30** |
| Aspect Ratio | 23 | 8 | 6 | 5 | 4 | 4 |
| Area | 1387 | 345 | 155 | 89 | 58 | 41 |
| Dmin | 323 | 83 | 39 | 24 | 16 | 12 |
| Dmax | 337 | 87 | 40 | 24 | 17 | 13 |
| Roundness | 38 | 12 | 7 | 6 | 5 | 4 |
| Circularity | 27 | 9 | 6 | 5 | 4 | 4 |
|  |  |  |  |  |  |  |
|  | **% Parameter Change** | | | | | |
| **Lateral** | **5** | **10** | **15** | **20** | **25** | **30** |
| Aspect Ratio | 78 | 22 | 12 | 8 | 6 | 5 |
| Area | 1672 | 421 | 189 | 108 | 70 | 50 |
| Dmin | 421 | 108 | 50 | 29 | 20 | 15 |
| Dmax | 391 | 100 | 46 | 28 | 19 | 14 |
| Roundness | 61 | 18 | 10 | 7 | 6 | 5 |
| Circularity | 43 | 13 | 8 | 6 | 5 | 4 |
|  |  |  |  |  |  |  |
| ***D. baroemoenensis*** | **% Parameter Change** | | | | | |
| **Umbilical** | **5** | **10** | **15** | **20** | **25** | **30** |
| Aspect Ratio | 30 | 10 | 6 | 5 | 4 | 4 |
| Area | 988 | 249 | 113 | 65 | 43 | 31 |
| Dmin | 274 | 71 | 33 | 20 | 14 | 11 |
| Dmax | 244 | 64 | 30 | 18 | 13 | 10 |
|  |  |  |  |  |  |  |
|  | **% Parameter Change** | | | | | |
| **Lateral** | **5** | **10** | **15** | **20** | **25** | **30** |
| Aspect Ratio | 103 | 28 | 15 | 10 | 7 | 6 |
| Area | 1225 | 309 | 139 | 80 | 52 | 37 |
| Dmin | 366 | 94 | 44 | 26 | 18 | 14 |
| Dmax | 311 | 80 | 38 | 27 | 16 | 12 |
